# Pigmented Cells in the Pineal Gland of Female Viscacha (*Lagostomus maximus maximus*): A Histochemical and Ultrastructural Study

**DOI:** 10.1155/2017/7492960

**Published:** 2017-12-17

**Authors:** Fabricio Ivan Busolini, Graciela Beatriz Rodríguez, Verónica Palmira Filippa, Fabian Heber Mohamed

**Affiliations:** ^1^Consejo Nacional de Investigaciones Científicas y Técnicas (CONICET), 5700 San Luis, Argentina; ^2^Histología, Facultad de Química, Bioquímica y Farmacia, Universidad Nacional de San Luis, Avenida Ejercito de los Andes 950, Bloque I, Piso No. 1, 5700 San Luis, Argentina; ^3^Parasitología, Facultad de Química, Bioquímica y Farmacia, Universidad Nacional de San Luis, Avenida Ejercito de los Andes 950, Bloque I, Piso No. 1, 5700 San Luis, Argentina

## Abstract

The presence of pigment has been demonstrated in different nervous structures such as those of retina, *substantia nigra*, and *locus coeruleus*. These pigments have also been described in the pineal gland of different mammal species. Histochemical and ultrastructural studies of the pineal gland of female viscacha (*Lagostomus maximus maximus*) were performed to analyze the presence of pigmented cells under natural conditions and to evaluate a probable relation between pigment content and glandular activity during pregnancy. The following techniques were applied: hematoxylin-eosin, phosphotungstic acid-hematoxylin, Masson-Fontana silver, DOPA histochemistry, Schmorl's reaction and toluidine blue. Estradiol and progesterone serum levels were determined by RIA. The ultrastructural features of the pineal pigment granules were also analyzed. Pigment granules were observed in a random distribution, but the pigmented cells were frequently found near blood vessels. The pineal pigment was histochemically identified as melanin. Differences in the amount of pigmented cells were found between pregnant and nonpregnant viscachas. The ultrastructural analysis revealed the presence of premelanosomes and melanosomes. Estradiol and progesterone levels vary during pregnancy. In conclusion, the changes in the amount of pigment content and hormone levels may indicate that the pineal gland of female viscacha is susceptible to endocrine variations during pregnancy.

## 1. Introduction

Melanin, a brownish-black pigment, is produced by the skin melanocytes which are derived from the neural crest and constitute the second most abundant cell in the epidermis [1, 2]. Its best-known function is to protect the skin against the harmful effects of the ultraviolet radiation [3–5]. Melanin plays critical functions most easily appreciated in furred mammals. These functions include thermal insulation, mimicry and camouflage (e.g., a seasonal change of coat colour occurs in the arctic fox), social and sexual communication (involving visual stimuli and odorant dispersal), and sensory perception like whiskers [1].

The presence of pigment has been demonstrated in different nervous structures such as those of retina, *substantia nigra*, and *locus coeruleus* [6–8]. In properly functioning nervous structures, the pigment is called neuromelanin [9]. The function of endogenous mediators of oxidative mechanisms among others has been indicated [10]. However, the role of pigment in these nervous structures has not been clearly demonstrated.

Pigments have been described in the pineal gland of different mammals such as bovines [11, 12], chinchilla [13], horse [14], bat [15, 16], dog [17], cat [18], sheep [19, 20], and humans [21–23]. However, the biological significance of these pigments in the pineal gland has not yet been established.

The viscacha (*Lagostomus maximus maximus*), the largest member of the Chinchillidae family, inhabits the southern hemisphere from Paraguay through central Argentina [24]. This rodent exhibits a nocturnal behaviour, lives in extensive burrows, and emerges at dawn and dusk to feed. Due to restricted light exposition, the viscacha constitutes an interesting model for studying the pineal gland and the processes in which it is involved. Female viscachas usually have an estrous period in early autumn and get pregnant during winter, although pregnant females have been found in others seasons [25]. The gestation period lasts approximately 154 days, and it ends in spring when the mother and offsprings have greater probabilities of survival [26]. In our laboratory, morphological studies of the male viscacha pineal gland have been carried out [27–29]. However, the female viscacha pineal gland has been scarcely studied [30].

The purpose of the present study is to analyze the presence of pigmented cells in the pineal gland of female viscachas under natural conditions and to evaluate a probable relation between pigment content and glandular activity during pregnancy.

## 2. Materials and Methods

### 2.1. Experimental Design

Adult female viscachas, weighing 2–4 kg, were captured in their habitat near San Luis, Argentina (33°20′ south latitude, 769 m altitude). Solar irradiation values, expressed as heliophany, and seasonal mean values of precipitation and temperature were provided by the Servicio Meteorológico Nacional (Table 1).

In this research, 16 female viscachas (12 pregnant and 4 nonpregnant) were used. The age and reproductive condition of viscachas were carefully assessed on the bases of (a) body weight [31] and (b) sexual maturity estimated by the ovaries histological analysis. Additionally, the uterine horns were examined to evaluate the presence of embryos, foetuses, and placental scars. The viscachas captured in summer (February) were nonpregnant. All females captured from early autumn to spring (April–September) were pregnant. According to the number and size of embryos or foetuses, pregnancy stages were classified as early pregnancy (April) with two or more 1–3 cm embryos, mid pregnancy (July) with two 9–11 cm foetuses, or late pregnancy (September) with two foetuses >19 cm. This classification was established according to previous reports in our laboratory [32–34].

After being captured, the animals were immediately taken to the laboratory, anesthetized with a combination of ketamine (Ketamina 50; Holliday-Scott®, Buenos Aires, Argentina) and xylazine (Pharmavet® S.A., Santa Fe, Argentina) at a dose of 12 and 0.4 mg/kg, respectively. The blood was collected by cardiac puncture for the evaluation of serum hormone concentration. The viscachas were quickly sacrificed by intracardiac injection of Euthanyle (0.25 ml/kg body weight, sodium pentobarbital, sodium diphenylhydantoin, Brouwer S.A.). The brains were exposed, and the pineal gland was immediately removed and used for light and electron microscopy.

The experimental design was approved by the local ethics committee and was in agreement with the guidelines of the National Institute of Health (NIH, USA) for the use of experimental animals. Moreover, the Biodiversity Control Area of the Environmental Ministry of San Luis (Argentina) approved a study protocol to carry out scientific research in the province (resolution N° 47-PBD-2015).

### 2.2. Light Microscopy

The samples were fixed in Bouin's fluid, embedded in paraffin, serially sectioned in the horizontal plane at 4 *μ*m, and stained with hematoxylin-eosin (H-E) and phosphotungstic acid-hematoxylin (PTAH). The Masson-Fontana silver and the Schmorl histochemical techniques for melanin pigments determination were also performed. For the Masson-Fontana silver method, the slides were deparaffinised in xylene, hydrated through decreasing concentrations of ethanol, and treated with 2.5% amoniacal silver nitrate solution in a 56°C oven for 2 h. After this procedure, the slides were thoroughly rinsed in distilled water, toned in 0.2% gold chloride for 1 min, rinsed in distilled water, treated with 5% sodium thiosulfate for 5 minutes, rinsed in distilled water, and counterstained with nuclear red fast solution for 2 min. Finally, the slides were dehydrated in an increasing concentration of ethanol, cleared in xylene, and mounted with Entellan® (Merck, Darmstadt, Germany). For Schmorl's reaction, slides were deparaffinised, hydrated, and treated with Schmorl working solution (ferric chloride 1% aqueous, 37.5 ml, potassium ferricyanide 1% aqueous 5 ml, and distilled water 7.5 ml) for 10 to 20 min. Then, the slides were rinsed in tap water, treated with 1% acetic acid for 5 min, and counterstained with Van Gieson's stain. Finally, the slides were dehydrated, cleared in xylene, and mounted using a synthetic mounting medium. To verify the histochemical nature of the pigments, the slides were treated with 0.5% acidified potassium permanganate (30 min) and after that with 1% oxalic acid (5–10 min) to obtain the melanin bleach. The hydrogen peroxide treatment was also carried out. The sections were examined using an Olympus BX-40 light microscope.

### 2.3. DOPA Histochemistry

The samples were fixed in 2% glutaraldehyde for 6 h and then sliced. These slices were placed in a 0.1% DL-DOPA solution (3,4-dihydroxyphenylanine, Sigma, in 0.1 M phosphate buffer at pH 7.4) and stored at 4°C in the refrigerator overnight. Next morning, they were incubated for 1-2 h at 37°C in a fresh solution made up with the same composition. After this treatment, the slices were fixed in Bouin's fluid, processed for light microscopy, embedded in paraffin, and sectioned (4 *μ*m). Labeling was assessed using an Olympus BX-40 light microscope.

### 2.4. Serum Levels of Estradiol and Progesterone

Blood samples were incubated at 37°C for 30 min in a water bath, centrifuged at 5000*g* for 10 min, and the serum removed. Serum estradiol and progesterone levels were quantified using the RIA Estradiol (A21854, Immunotech s.r.o., Prague, Czech Republic) and RIA Progesterone (IM1188, Immunotech s.r.o., Prague, Czech Republic) kits from Beckman Coulter®, respectively. These tests were competitive radioimmunoassays for *in vitro* use.

### 2.5. Electron Microscopy

For electron microscopy, the pineal glands were fixed “in situ” with formaldehyde-glutaraldehyde in phosphate buffer [35] for 10 min, removed and placed in the same fixative for an additional 6-hour period at room temperature, post fixed in cold 2% OsO_4_ for 12 h, dehydrated in acetone, and embedded in Spurr's resin. One-micrometer-thick sections were obtained with a Porter Blum ultramicrotome and dyed with toluidine blue for light microscopy. Ultrathin sections were stained with uranyl acetate and lead citrate [36] and were observed under a Siemens Elmiskop I electron microscope.

### 2.6. Morphometric Analysis

The pigmented cells were counted in pregnant and nonpregnant viscachas. For this purpose, four pineal glands per group were analyzed. Three regularly spaced serial tissue sections (50 *μ*m each) from each gland were used. For each section, 10 microscopic fields were randomly selected throughout the pineal gland. Each field contained between 90 and 110 cells. Per field, the percentage of pigmented cells was obtained according to the following formula: A/(A + B) × 100. The number of pigmented cells (A) and of remaining parenchymal cells (B, pinealocytes and interstitial cells) was counted. A 40x objective was used for the observations.

### 2.7. Statistical Analysis

The results were expressed as mean ± standard error of the mean (SEM). The groups were analysed using the Kruskal-Wallis test. A value of *p* < 0.05 was considered statistically significant. InfoStat software version 2011 [37] was used for this analysis.

## 3. Results

**Figure 1:**
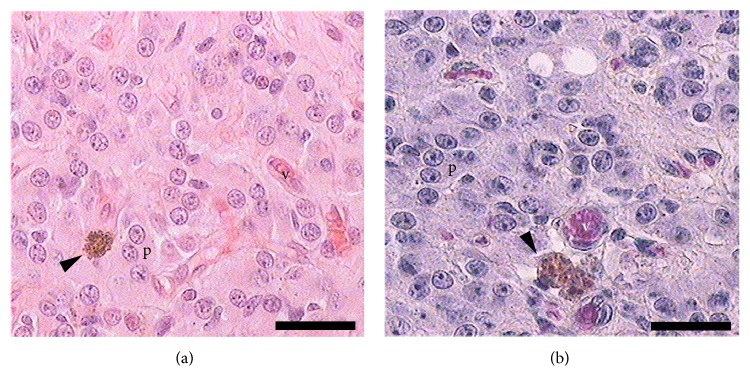
Image of female pineal gland in early pregnancy. (a) The photomicrograph shows a pigmented cell (arrowhead) surrounded by pinealocytes (p). Some blood vessels are observed (v). Hematoxylin-eosin. (b) Pigmented cells (arrowhead) in close association with blood vessels (v). Modified phosphotungstic acid-hematoxylin. Scale bars: (a) and (b) 25 *μ*m.

The pineal gland of female viscacha presented an oval-like morphology, elongated, with a stalk connected to the brain. Two different regions in the parenchyma were observed (proximal region and distal region). The light microscopy study revealed the presence of pigments in the pineal gland for all the groups. In sections stained with H-E and PTAH, the pigmented cells were ovoid or rounded in shape, and they appeared light to dark brown in colour. They exhibited a random distribution throughout the gland, but they were frequently found near blood vessels. Some of these cells showed a higher amount of pigment content in their cytoplasm (Figure 1).

**Figure 2:**
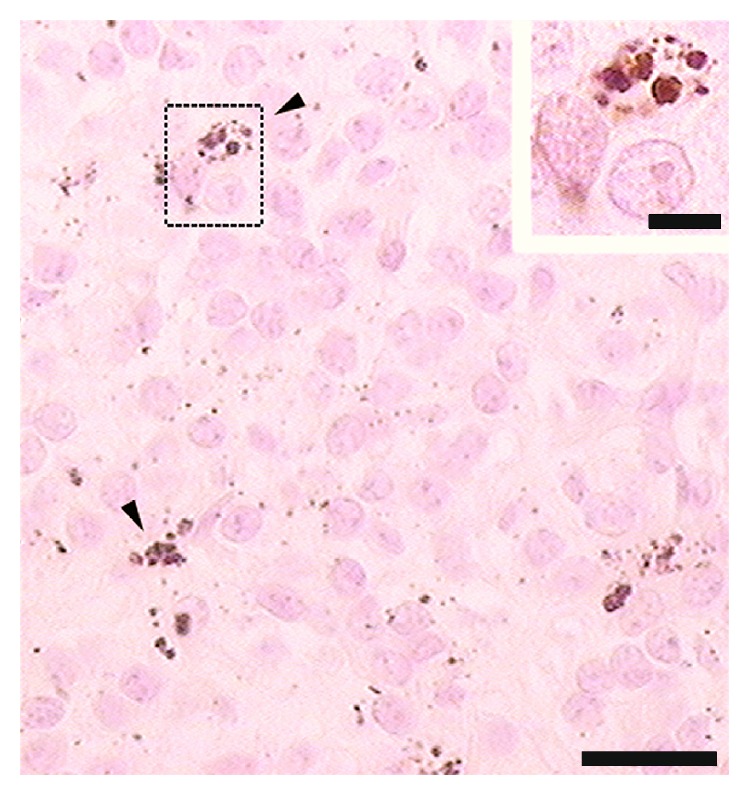
Image of female pineal gland in mid pregnancy. A strong argentaffin reaction is exhibited in pigmented cells (arrowheads) and in extracellular space. Abundant pigment granules are dispersed throughout the parenchyma. The inset shows the argentaffin reaction with greater detail. Masson-Fontana silver method, counterstained with nuclear fast red. Scale bar: 25 *μ*m. Inset scale bar: 5 *μ*m.

The Masson-Fontana silver method for melanin resulted positive and the pigments manifested a strong argentaffin reaction. In the extracellular space, pigment granules were abundant and dispersed throughout the parenchyma (Figure 2). The pigment granules of control slides were bleached by the treatment with potassium permanganate-acid oxalic and hydrogen peroxide, which identified the pigment histochemically as melanin.

**Figure 3:**
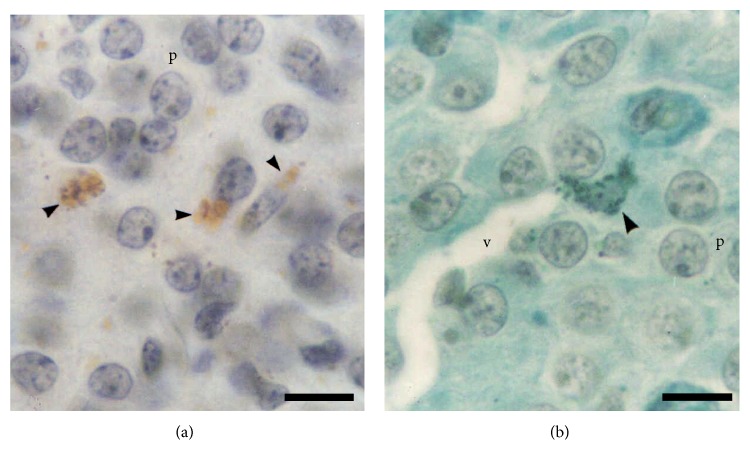
Image of female pineal gland in late pregnancy. (a) A positive DOPA histochemistry reaction is observed in the pigmented cells (arrowheads) in close relation to the pinealocytes (p). Nuclear counterstain: haematoxylin. (b) The photomicrograph shows a pigmented cell (arrowhead) near a blood vessel (v). Schmorl's reaction, counterstain: Van Gieson. Scale bars: (a) and (b) 10 *μ*m.

**Figure 4:**
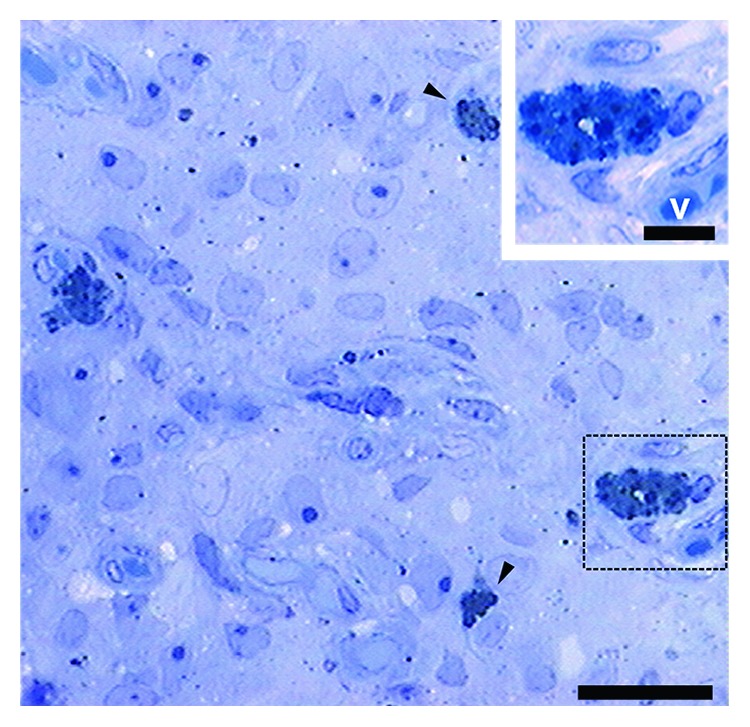
Image of female pineal gland in mid pregnancy. The photomicrograph shows a semithin section with numerous pigmented cells (arrowheads) in close relation to blood vessels (v). The inset shows the pigmented cell in close relation to the blood vessel in greater magnification. Toluidine blue. Scale bar: 25 *μ*m. Inset scale bar: 5 *μ*m.

The DOPA histochemistry and Schmorl's reaction were also positive. In DOPA histochemistry, pigments were brown (Figure 3(a)), whereas in Schmorl's reaction, pigments appeared green in colour (Figure 3(b)). Semithin section stained with toluidine blue exhibited numerous pigmented cells in close relation to blood vessels (Figure 4).

**Figure 5:**
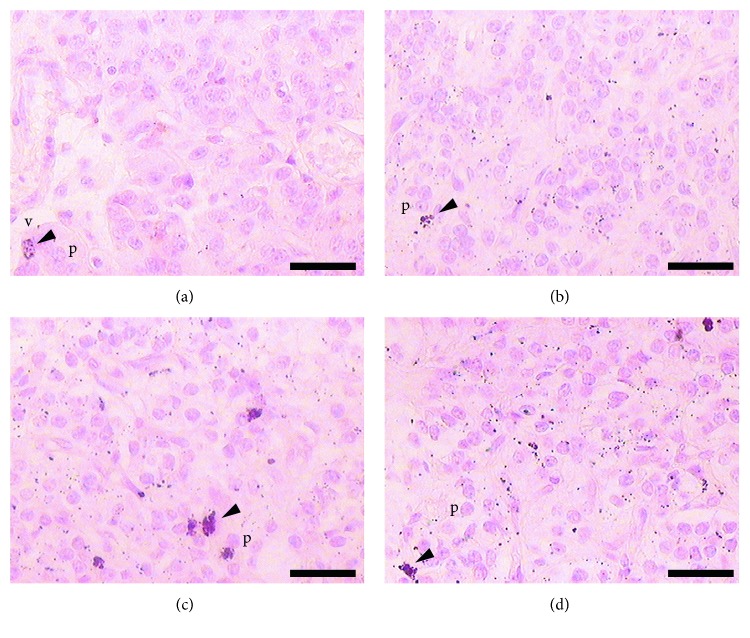
Photomicrograph of pigmented cells (arrowhead) in each pregnancy stage. (a) Nonpregnancy, (b) early pregnancy, (c) mid pregnancy, and (d) late pregnancy. Pinealocytes (p). Masson-Fontana silver method, counterstained with nuclear fast red. (a–d) scale bars: 25 *μ*m.

Variations in the amount of pineal pigments related to serum hormone levels were found between pregnant and nonpregnant viscachas. The pregnant ones presented a higher amount of pigments distributed within the parenchyma (Figure 5). Regarding the number of pigment cells in each stage, females in mid pregnancy exhibited a higher number of pigmented cells, followed by females in late pregnancy and early pregnancy. The nonpregnant viscachas showed the lowest number of pigmented cells in all the groups studied (Table 2).

**Figure 6:**
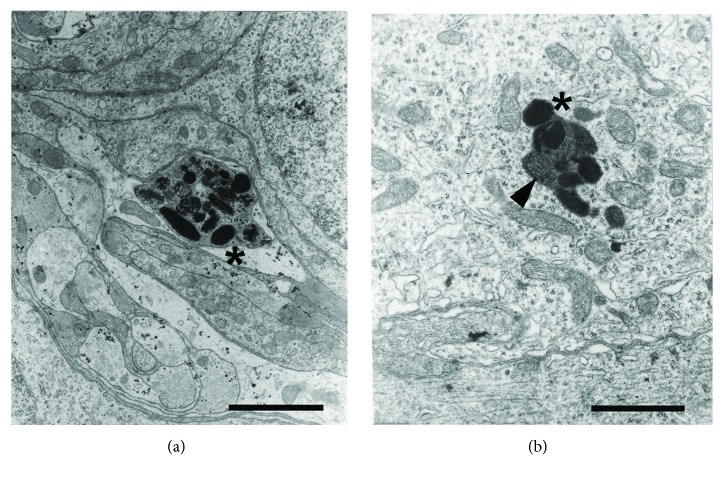
Electron micrograph of female pineal gland in mid pregnancy. (a) Accumulation of pigment granules in different stages of melanogenesis (∗). (b) Pigment granules (∗) within a lysosome-like dense body (arrowhead). Scale bars: (a) 4 *μ*m and (b) 2 *μ*m.

**Figure 7:**
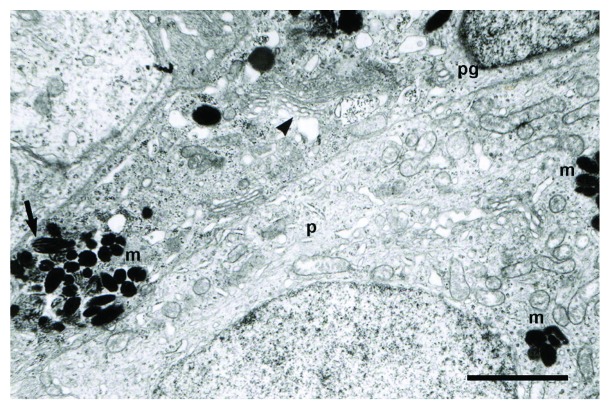
Ultrastructural features of female pineal gland in mid pregnancy. The electron micrograph shows a large amount of premelanosomes (arrow) and melanosomes (m) within a pinealocyte (p) and a pigmented cell (pg). The arrowhead indicates a well-developed Golgi complex in the pigmented cell. The pinealocyte exhibited a large amount of mitochondria in the cytoplasm. Scale bars: 3 *μ*m.

The electron microscopy study revealed the presence of cells containing electron-dense granules, whose ultrastructural features corresponded to premelanosomes and melanosomes. Premelanosomes and melanosomes were identified in pigmented cells, pinealocytes, and interstitial cells. Several melanogenesis stages were observed (Figure 6(a)). In the pinealocytes, the melanosomes were often found within lysosome-like dense bodies (Figure 6(b)). The ultrastructure of the pigmented cells exhibited an ovoid or rounded nucleus in shape, with heterochromatin dispersed in the nuclear matrix. The cytoplasm presented abundant mitochondria and a well-developed Golgi complex. The cytoplasm of pigmented cells was characterized by the presence of variable size granules with an electron-dense content (Figure 7).

## 4. Discussion

The morphology of the mammal pineal gland presents great variability [19, 38, 39]. Although with no clearly established function, infrequent structures, such as synaptic ribbons and spherules, cilia, ciliary derivatives, brain sand, and melanin granules, constitute a regular feature. The occurrence of melanin has been described in the pineal gland of some mammal species, but the reports are scarce [11, 14, 16–20]. This suggests that melanin is not a constant characteristic in the pineal gland of mammals. The biological significance of this pigment in the gland has not been clearly determined. Nevertheless, the embryonic processes might explain the presence of melanin. The pineal gland and the retina are formed from the developing diencephalon. Some authors have indicated the presence of a few retinal antigens in the pinealocytes [40–43]. In our laboratory, the morphology of the viscacha retinal melanosomes has been described [44]. Here, the pineal melanosomes morphology exhibited a remarkable similarity in relation to the one previously reported in retina by Calderón et al. [44]. Thus, the pineal melanin might be a consequence of the homology amongst these organs.

In the present study, the histochemical and ultrastructural results demonstrate the presence of melanin in the female viscacha pineal gland. The greatest amount of pigment granules were identified in the pigmented cells. Gil et al. [30] reported that pigment content was scarcely observed in pinealocytes, especially dark pinealocytes. In our study, a low content of pigments were found in pinealocytes as well as in the interstitial cells. However, in the extracellular space, pigment granules were abundant and dispersed throughout the parenchyma, especially in mid and late pregnancy.

The morphology, histochemical properties, and ultrastructure of the pigmented cells may indicate that the melanogenesis takes place in these cells, and then, the melanin is transferred to pinealocytes and interstitial cells. This process of melanin transference has already been suggested in cat [18] and bat [16]. In female viscacha, melanin granules within lysosome-like dense bodies were also observed. Calvo et al. [18] described similar structures in cat pineal gland.

The amount of melanin in relation to sex varies according to the species. In dog [17], cat [18], sheep [19, 20], bat [16], and bovine [12], significant differences were not reported. In contrast, the pigmented cells number varied significantly according to sex in viscacha. The pigments were exceptionally observed in adult and immature male viscachas [29]. However, in the present study, the pineal pigments were evident in female viscachas.

Previous studies have indicated that the adult male viscacha is a photoperiod-dependent seasonal breeder [29, 45–48]. Under these conditions, the photoperiod and the retinal-pineal axis play an important role. In contrast, in female viscachas, the relevance of the photoperiod and the pineal function remains unclear.

Estrogens and progesterone are known to be able to induce melanin biosynthesis [49, 50]. In female viscacha, estradiol and progesterone levels are subject to variations during pregnancy. Thus, the changes in the amount of pineal melanin seems to indicate a direct relationship between pigment content and gonadal hormone levels during pregnancy, when estradiol and progesterone levels remain elevated for a long time.

In conclusion, we suggest that the pineal gland of female viscacha may be more susceptible to endocrine cues during pregnancy and less subject to the environmental photoperiod, as in the case of the male viscacha. However, further studies are necessary to confirm this relationship.

## Figures and Tables

**Table 1:** Seasonal environmental conditions.

Parameters	Summer	Autumn	Winter	Spring
Heliophany (h)	9.38	7.09	6.82	9.09
Precipitation (mm)	90	27	11	58.5
Temperature (°C)	22	13	12	19.66

**Table 2:** Variations in the number of pigmented cells and serum hormone levels between nonpregnant and pregnant viscachas.

Parameters	Nonpregnant	Early pregnancy	Mid pregnancy	Late pregnancy
Number of pigmented cells	0.76 ± 0.16	1.73 ± 0.18^a^	3.52 ± 0.35^b^	2.58 ± 0.12^c^
Estradiol (pg/ml)	18 ± 3.19	27.5 ± 2.5	75 ± 2.89^d^	24.25 ± 2.17
Progesterone (ng/ml)	0.72 ± 0.11	4.64 ± 0.95^e^	53.75 ± 2.39^f^	18.61 ± 2.25^g^

The values are expressed as mean ± SEM (*n* = 4). Number of pigmented cells: ^a^*p* < 0.05, early pregnancy versus nonpregnant; ^b^*p* < 0.05, mid pregnancy versus nonpregnant and early pregnancy; ^c^*p* < 0.05, late pregnancy versus nonpregnant and early pregnancy. Estradiol: ^d^*p* < 0.05, mid pregnancy versus early pregnancy, late pregnancy, and nonpregnant. Progesterone: ^e^*p* < 0.05, early pregnancy versus nonpregnant, ^f^*p* < 0.05, mid pregnancy versus early pregnancy, late pregnancy, and nonpregnant; ^g^*p* < 0.05, late pregnancy versus early pregnancy and nonpregnant. The significant differences were determined by Kruskal-Wallis test.

## References

[1] Slominski A., Tobin D. J., Shibahara S., Wortsman J. (2004). Melanin pigmentation in mammalian skin and its hormonal regulation. *Physiological Reviews*.

[2] Nordlund J. J. (2007). The melanocyte and the epidermal melanin unit: an expanded concept. *Dermatologic Clinics*.

[3] Brenner M., Hearing V. J. (2008). The protective role of melanin against UV damage in human skin. *Photochemistry and Photobiology*.

[4] Herrling T., Jung K., Fuchs J. (2008). The role of melanin as protector against free radicals in skin and its role as free radical indicator in hair. *Spectrochimica Acta Part A: Molecular and Biomolecular Spectroscopy*.

[5] D’Orazio J., Jarrett S., Amaro-Ortiz A., Scott T. (2013). UV radiation and the skin. *International Journal of Molecular Science*.

[6] Foley J. M., Baxter D. (1958). On the nature of pigment granules in the cells of the locus coeruleus and substantia nigra. *Journal of Neuropathology & Experimental Neurology*.

[7] Zecca L., Tampellini D., Gerlach M., Riederer P., Fariello R. G., Sulzer D. (2001). Substantia nigra neuromelanin: structure, synthesis, and molecular behaviour. *Molecular Pathology*.

[8] Zecca L., Zucca F. A., Wilms H., Sulzer D. (2003). Neuromelanin of the substantia nigra: a neuronal black hole with protective and toxic characteristics. *Trends in Neurosciences*.

[9] Graham D. G. (1979). On the origin and significance of neuromelanin. *Archives of Pathology & Laboratory Medicine*.

[10] Fedorow H., Tribl F., Halliday G., Gerlach M., Riederer P., Double K. L. (2005). Neuromelanin in human dopamine neurons: comparison with peripheral melanins and relevance to Parkinson’s disease. *Progress in Neurobiology*.

[11] Meyer-Arendt J., Santamarina E. (1956). Identification of melanin in the bovine pineal gland. *Acta Histochemica*.

[12] Santamarina E. (1958). Melanin pigmentation in bovine pineal gland and its possible correlation with gonadal function. *Canadian Journal of Biochemistry and Physiology*.

[13] Matsushima S., Reiter R. J., Hess M. (1975). Comparative ultrastructural studies of the pineal gland of rodents. *Comparative Ultrastructural Studies of the Pineal Gland of Rodents*.

[14] Cozzi B. (1986). Cell types in the pineal gland of the horse: an ultrastructural and immunocytochemical study. *The Anatomical Record*.

[15] Bhatnagar K. P. (1988). Ultrastructure of the pineal body of the common vampire bat, *Desmodus rotundus*. *The American Journal of Anatomy*.

[16] Bhatnagar K. P., Hilton F. K. (1994). Observations on the pineal gland of the big brown bat, *Eptesicus fuscus*: possible correlation of melanin intensification with constant darkness. *The Anatomical Record*.

[17] Calvo J., Boya J., García-Mauriño J. E., Lopez-Carbonell A. (1988). Structure and ultrastructure of the pigmented cells in the adult dog pineal gland. *Journal of Anatomy*.

[18] Calvo J. L., Boya J., García-Mauriño J. E., Rancaño D. (1992). Presence of melanin in the cat pineal gland. *Acta Anatomica*.

[19] Regodón S., Franco A. J., Gazquez A., Redondo E. (1998). Presence of pigment in the ovine pineal gland during embryonic development. *Histology and Histopathology*.

[20] Redondo E., Regodon S., Masot J., Gázquez A., Franco A. (2003). Postnatal development of female sheep pineal gland under natural inhibitory photoperiods: an immunocytochemical and physiological (melatonin concentration) study. *Histology and Histopathology*.

[21] Møller M. (1974). The ultrastructure of the human fetal pineal gland. I. Cell types and blood vessels. *Cell and Tissue Research*.

[22] Min K. W., Seo I. S., Song J. (1987). Postnatal evolution of the human pineal gland. An immunohistochemical study. *Laboratory Investigation*.

[23] Koshy S., Vettivel S. (2001). Melanin pigments in human pineal gland. *Journal of the Anatomical Society of India*.

[24] Redford K. H., Eisenberg J. P. (1992). *Mammals of the Neotropics. The shouthern Cone. Volume 2*.

[25] Jackson J. E. (1986). Determinación de la edad en la vizcacha (*Lagostomus maximus*) en base al peso del cristalino. *Vida Silvestre Neotropical*.

[26] Weir B. J. (1971). The reproductive physiology of the plains viscacha, *Lagostomus maximus*. *Journal of Reproduction and Fertility*.

[27] Domínguez S., Piezzi R. S., Scardapane L., Guzmán J. A. (1987). A light and electron microscopic study of the pineal gland of the viscacha (*Lagostomus maximus maximus*). *Journal of Pineal Research*.

[28] Cernuda-Cernuda R., Piezzi R. S., Domínguez S., Alvarez-Uría M. (2003). Cell populations in the pineal gland of the viscacha (*Lagostomus maximus*). Seasonal variations. *Histology and Histopathology*.

[29] Busolini F. I., Rosales G. J., Filippa V. P., Mohamed F. H. (2017). A seasonal and age-related study of interstitial cells in the pineal gland of male viscacha (*Lagostomus maximus maximus*). *The Anatomical Record*.

[30] Gil E., Calderon C., Pelzer L. (2005). Morphological and biochemical study of the pineal gland of pregnant and non-pregnant female vizcachas (*Lagostomus maximus maximus*). *Neuro Endocrinology Letters*.

[31] Llanos A. C., Crespo J. A. (1952). Ecología de la vizcacha (*Lagostomus maximus maximus* Blainv.) en el nordeste de la provincia de Entre Ríos. *Revista de investigaciones agricolas*.

[32] Filippa V. P., Mohamed F. H. (2010). Morphological and morphometric changes of pituitary lactotrophs of viscacha (*Lagostomus maximus maximus*) in relation to reproductive cycle, age, and sex. *The Anatomical Record*.

[33] Filippa V. P., Mohamed F. H. (2010). The pituitary of non-pregnant and pregnant viscachas (*Lagostomus maximus maximus*): a comparative study by immunohistochemistry and morphometric analysis. *Zoology*.

[34] Gil E., Forneris M., Domínguez S. (2007). Morphological and endocrine study of the ovarian interstitial tissue of viscacha (*Lagostomus maximus maximus*). *The Anatomical Record*.

[35] Karnovsky M. J. (1965). A formaldehyde-glutaraldehyde fixative of high osmolality for use in electron microscopy. *The Journal of Cell Biology*.

[36] Millonig G. (1961). A modified procedure for lead staining of thin sections. *The Journal of Cell Biology*.

[37] Di Rienzo J. A., Casanoves F., Balzarini M. G. (2011). InfoStat versión 2011. *Grupo InfoStat*.

[38] Bhatnagar K. P. (1992). The ultrastructure of mammalian pinealocytes: a systematic investigation. *Microscopy Research & Technique*.

[39] Karasek M., Reiter R. J. (1992). Morphofunctional aspects of the mammalian pineal gland. *Microscopy Research & Technique*.

[40] Huang S. K., Klein D. C., Korf H. W. (1992). Immunocytochemical demonstration of rod-opsin, S-antigen, and neuron-specific proteins in the human pineal gland. *Cell and Tissue Research*.

[41] Korf H. W., Sato T., Oksch A. (1990). Complex relationships between the pineal organ and the medial habenular nucleus-pretectal region of the mouse as revealed by S-antigen immunocytochemistry. *Cell and Tissue Research*.

[42] Korf H. W., White B. H., Schaad N. C., Klein D. C. (1992). Recoverin in pineal organs and retinae of various vertebrate species including man. *Brain Research*.

[43] Schomerus C., Ruth P., Korf H. W. (1994). Photoreceptor-specific proteins in the mammalian pineal organ: immunocytochemical data and functional considerations. *Acta Neurobiologiae Experimentalis*.

[44] Calderón C., Mohamed F., Muñoz E. (2002). Daily morphological variations in the viscacha (*Lagostomus maximus maximus*) retina. Probable local modulatory action of melatonin. *The Anatomical Record*.

[45] Aguilera-Merlo C., Muñoz E., Domínguez S., Scardapane L., Piezzi R. (2005). Epididymis of viscacha (*Lagostomus maximus maximus*): morphological changes during the annual reproductive cycle. *The Anatomical Record*.

[46] Acosta M., Mohamed F. (2011). Effect of the photoperiod and administration of melatonin on folliculostellate cells of the pituitary pars distalis of adult male viscacha (*Lagostomus maximus maximus*). *Acta Histochemica*.

[47] Chaves E. M., Aguilera-Merlo C., Cruceño A. (2012). Seasonal morphological variations and age-related changes of the seminal vesicle of viscacha (*Lagostomus Maximus Maximus*): an ultrastructural and immunohistochemical study. *The Anatomical Record*.

[48] Filippa V. P., Rosales G. J., Cruceño A. M., Mohamed F. H. (2015). Androgen receptors expression in pituitary of male viscacha in relation to growth and reproductive cycle. *International Journal of Endocrinology*.

[49] Hall P. P. (1969). The influence of hormones on melanogenesis. *The Australasian Journal of Dermatology*.

[50] Maeda K., Naganuma M., Fukuda M., Matsunaga J., Tomita Y. (1996). Effect of pituitary and ovarian hormones on human melanocytes in vitro. *Pigment Cell Research*.

